# Unprecedented drought over tropical South America in 2016: significantly under-predicted by tropical SST

**DOI:** 10.1038/s41598-017-05373-2

**Published:** 2017-07-19

**Authors:** Amir Erfanian, Guiling Wang, Lori Fomenko

**Affiliations:** Department of Civil and Environmental Engineering and Center for Environmental Sciences and Engineering, University of Connecticut, Storrs, Connecticut USA

## Abstract

Tropical and sub-tropical South America are highly susceptible to extreme droughts. Recent events include two droughts (2005 and 2010) exceeding the 100-year return value in the Amazon and recurrent extreme droughts in the Nordeste region, with profound eco-hydrological and socioeconomic impacts. In 2015–2016, both regions were hit by another drought. Here, we show that the severity of the 2015–2016 drought ("2016 drought" hereafter) is unprecedented based on multiple precipitation products (since 1900), satellite-derived data on terrestrial water storage (since 2002) and two vegetation indices (since 2004). The ecohydrological consequences from the 2016 drought are more severe and extensive than the 2005 and 2010 droughts. Empirical relationships between rainfall and sea surface temperatures (SSTs) over the tropical Pacific and Atlantic are used to assess the role of tropical oceanic variability in the observed precipitation anomalies. Our results indicate that warmer-than-usual SSTs in the Tropical Pacific (including El Niño events) and Atlantic were the main drivers of extreme droughts in South America, but are unable to explain the severity of the 2016 observed rainfall deficits for a substantial portion of the Amazonia and Nordeste regions. This strongly suggests potential contribution of non-oceanic factors (e.g., land cover change and CO2-induced warming) to the 2016 drought.

## Introduction

Greenhouse warming is anticipated to change drought outlook in the future climate^1–3^ and enhance drought severity in particular^4^. Tropical South America has been considered a drought hotspot in future climate projections due to its potential to respond drastically to excessive warming and drying^2, 3, 5, 6^. Even in the present-day climate, sub-tropical savannah areas of South America are facing severe water shortages and heightened risks of desertification due to extreme droughts^6^. In the Amazon basin, recurrent droughts over short time periods have been found to inhibit recovery of the region’s eco and hydro systems, increasing wildfire risk and tree mortality and accelerating ecosystem carbon emissions^7–13^. In the past decade (2005 to 2016) alone, tropical/subtropical South America has experienced several extreme drought events. In 2005, a severe drought in the Amazon, categorized as a 100-year event^9, 11, 14^, caused record-breaking annual wild fires and carbon emissions, leading to the first ever negative annual carbon balance recorded for the rainforest^9, 11^. Five years later, a stronger and more destructive drought hit Amazonia in 2010 and the recorded rainforest carbon balance was negative for the second time^11, 15^. The Northeastern Brazil (Nordeste) region also experienced extreme droughts in 2005, 2007, 2010, and 2012. The 2012 drought was the most extreme in the past several decades and caused widespread shortages in drinking/irrigation water and hydroelectric energy supply over this densely populated semi-arid region^6, 10, 16^.

Variability of the tropical oceans, including both the Pacific and Atlantic, has been considered the main driver for inter-annual variability of precipitation over the Amazon basin and Nordeste region^6, 8, 9, 11, 15, 17, 18^. In the Pacific, El Niño events are important predictors for severe droughts over these areas^19–21^. A warmer tropical Pacific perturbs the Walker circulation and weakens the trade winds. The weakening of the easterlies reduces convergence of moisture from the tropical Atlantic (the major supplier of moisture for tropical South America) to the continental interior. El Niño-induced perturbations in the Walker circulation, Hadley Cell and Rossby Waves also increase subsidence and suppress convection over the region^20, 22–24^. In the Atlantic, a warmer-than-usual tropical North Atlantic causes an anomalous northward shift of the ITCZ and weakens trade winds below the Equator subsequently reducing moisture convergence and weakening convection over the Amazon and Nordeste regions. This warming also causes an anomalous rising motion in the north which is coupled with an anomalous sinking motion over South America and the Southern Atlantic (by the Hadley circulation) further reducing rainfall over the subsidence zone^8, 12, 15, 18^. SST forcings from these two basins are responsible for two different types of regional droughts: El Niño-induced droughts during SON (pre-monsoon) and DJF (monsoon seasons), and droughts occurring in response to a warmer Tropical Atlantic typically during the dry season (May to September).

Anomalously warm tropical Pacific SSTs detected in early 2014 set the stage for an immense El Niño, yet the 2014 El Niño was less intense than predicted. However, the following year witnessed an El Niño that was comparable to the 1983 and 1998 events^25^. The tropical Pacific Ocean remained anomalously warm from 2014–2016, and a severe drought was expected to hit tropical South America in 2016^26^. Over the Nordeste region, anomalously warmer SSTs in the tropical Pacific and Atlantic were expected to intensify drought conditions that had been persisting since 2012^6, 10^; over the Amazon basin, preliminary analysis of the 2015–2016 wet season confirmed a severe drought condition^17^.

In this paper, we examine the spatial extent, temporal evolution, and severity of the 2016 South American drought and its manifestation in terrestrial water storage (TWS) and vegetation greenness based on gridded observational data sets. The datasets used include multiple gauge-derived precipitation datasets, satellite-derived TWS data from Gravity Recovery and Climate Experiment (GRACE)^27, 28^, Normalized Difference Vegetation Index (NDVI) and Enhanced Vegetation Index (EVI) data from Moderate Resolution Imaging Spectroradiometer (MODIS) sensors. We compare the 2016 drought with the record-breaking droughts of 2005 and 2010, as well as the two El Niño droughts of 1983 and 1998, and relate precipitation anomalies to tropical SST variability to assess SST-based drought predictions. The strong relationship between rainfall and SST in the adjacent tropical oceans has allowed statistical forecast models to skillfully predict precipitation in the region^8, 19, 29^. However, oceanic forcing is not the only driver for this regional climate. Disturbances and other processes over land, including deforestation, surface warming, and CO2 fertilization, can also influence the regional hydroclimate regime^7, 24, 30–33^. Here we developed a statistical model and implemented hypothesis testing to investigate how much of the observed precipitation deficits could be explained by anomalies of tropical SSTs.

## Results

**Figure 1:**
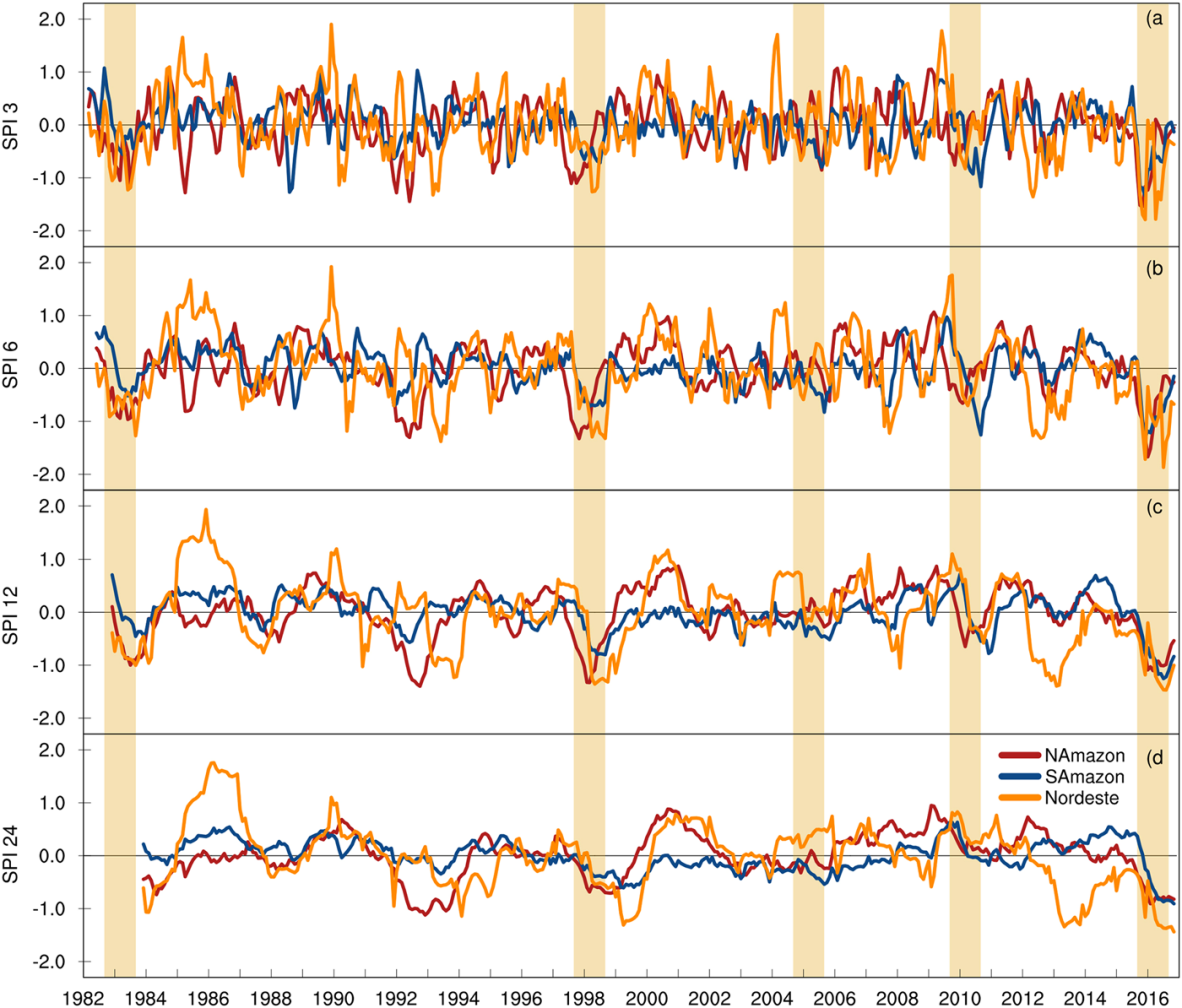
Time series of Standardized Precipitation Index (SPI). The entire Tropical/sub-Tropical South America is divided into South Amazon (SA), North Amazon (NA), and Nordeste (NORD) regions to construct the area-averaged time series (see Figure S1 for details). The 3, 6, 12, and 24-month SPIs shown in (**a**,**b**,**c** and **d**) respectively were calculated using the GPCC monthly precipitation data from 1982 to 2017. For all time scales, the 2016 SPIs over Nordeste and South Amazon are the largest seen in the analysis period. Figures created with: The NCAR Command Language (Version 6.3.0) [Software]. (2016). Boulder, Colorado: UCAR/NCAR/CISL/TDD. http://dx.doi.org/10.5065/D6WD3XH5.

The Standardized Precipitation Index (SPI) is used as a metric for the severity of rainfall deficits. Figure 1 presents the SPI time series for 3, 6, 12, and 24-month timescales averaged over the Nordeste region, North and South Amazonia (see Figure S1 for definition of these regions). All three regions had negative SPI values for the entire hydrological year of 2016. Over the two sub-regions of Amazonia, the 2016 drought peaked in the SON and DJF seasons with short term (3 and 6 month) SPI values close to the extreme drought level of −2 (Fig. 1a and b), which were record lows for the study period (1982–2017). For the Nordeste region, the SPI peaked once at the beginning (SON) and once at the end (MAM) of the 2016 wet season. The lowest SPI values over the South Amazon and Nordeste regions, for all timescales presented in Fig. 1, were recorded in 2016. Over North Amazon, the SPI values in 2016 are comparable with the record low values for the region during the 1992 and 1998 events. These statements are also valid for SPI analysis over the 1901–2017 period (Figure S2)–the SPIs calculated for 2016 show the worst drought ever recorded over South Amazon and Nordeste since 1901 with only two other droughts (1904 and 1916) reaching a similar level of severity.

**Figure 2:**
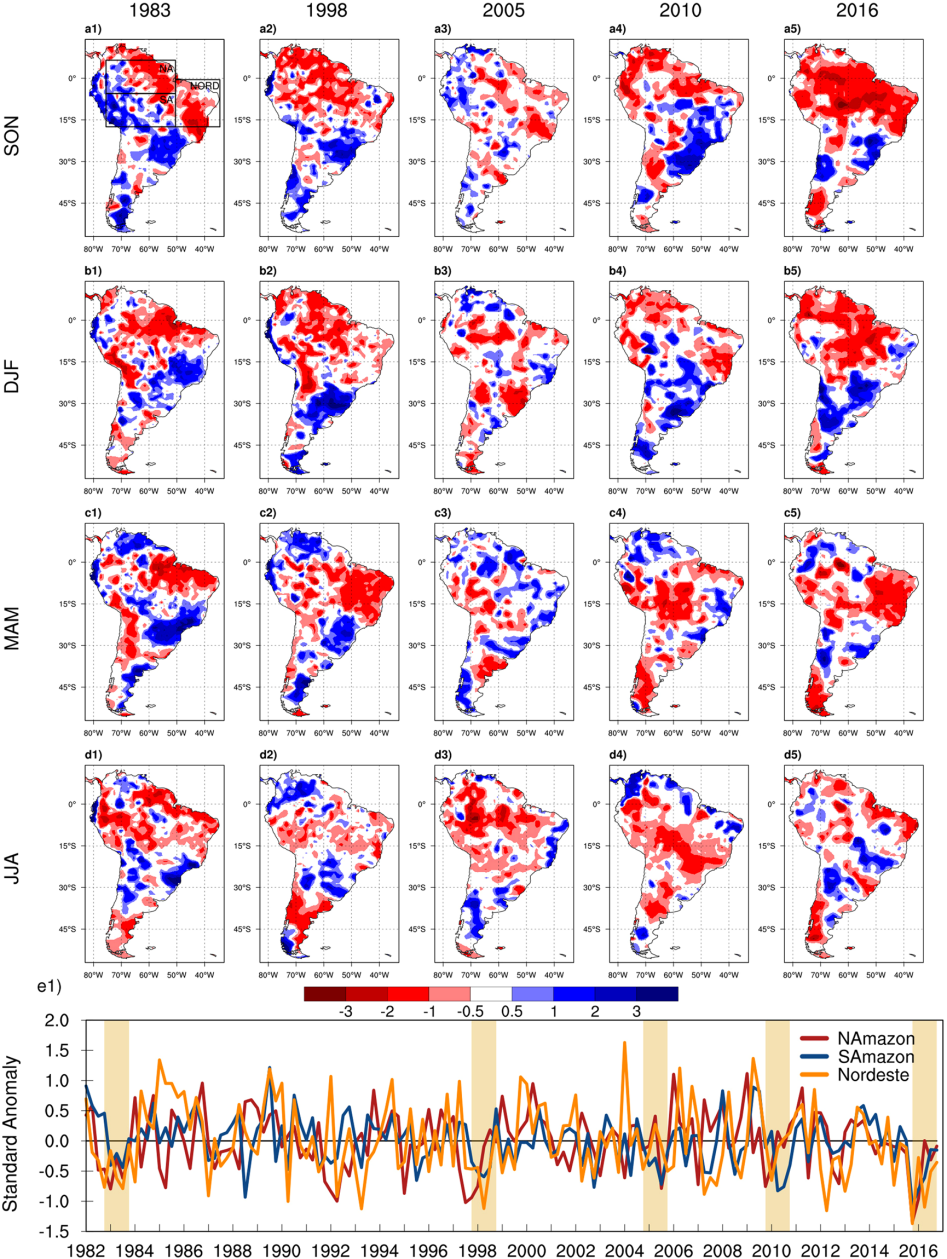
Spatial maps and time series of standardized precipitation anomalies (dimensionless) calculated for the 1982–2017 period. The contour plots present the seasonal rainfall anomalies for the five extreme droughts of 1983 (**a1** to **d1**), 1998 (**a2** to **d2**), 2005 (**a3** to **d3**), 2010 (**a4** to **d4**), and 2016 (**a5** to **d5**). The monthly time series (**e1**) were constructed by averaging the 3-month SPI over the North Amazon, South Amazon, and Nordeste regions (the box shown in **a1**) and the highlight strips denote the five extreme droughts represented in the spatial maps. Seasonal cycles for each year start from September of the previous year (e.g. SON 2004 for 2005 seasonal cycle). Figures created with: The NCAR Command Language (Version 6.3.0) [Software]. (2016). Boulder, Colorado: UCAR/NCAR/CISL/TDD. http://dx.doi.org/10.5065/D6WD3XH5.

The standardized anomalies of precipitation in each season (Fig. 2) indicate that the 2016 drought impacted the entirety of South America north of 20°S during the austral spring and summer. The center of the maximum rainfall deficit moves eastward from southern Amazon in SON (Fig. 2-a5) to northeastern Amazon in DJF (Fig. 2-b5) and the Nordeste region in MAM and JJA (Fig. 2-c5 and d5). The 2016 rainfall deficits during the SON and DJF seasons have similar spatial patterns as those of the 1983 and 1998 El Nino droughts, but are more extensive and more severe (Fig. 2). During the MAM season, the largest deficits are found over the Nordeste region.

The precipitation anomalies remained negative during JJA over much of the Amazon and Nordeste regions in 2016. Among the five droughts highlighted in Fig. 2, the rainfall standardized anomalies for the JJA season are largest in 2005 and 2010 (also see Figure S3). The similarities and distinctions among these highlighted extreme events are mostly rooted in the oceanic forcings driving them. The 2005 and 2010 droughts are mainly attributed to strong warm anomalies of tropical Atlantic SSTs extending and peaking during the dry season (May to September) resulting in the largest standardized anomalies being experienced over that period^8, 9, 11, 15, 18^. The 2010 wet season also had negative rainfall anomalies, which were attributed to the moderate El Niño of 2009^11, 15, 18^ (see Figure S4-e). The 2016 rainfall anomalies, however, peaked during the SON and DJF seasons in response to the strong El Niño of 2015–2016. In addition, the SSTs in the tropical Atlantic remained anomalously warm throughout 2016 (Figure S4-e). As a result, the rainfall deficits over much of Southern Amazon and Nordeste persisted throughout the dry season (JJA) despite the El Niño demise in late April.

**Figure 3:**
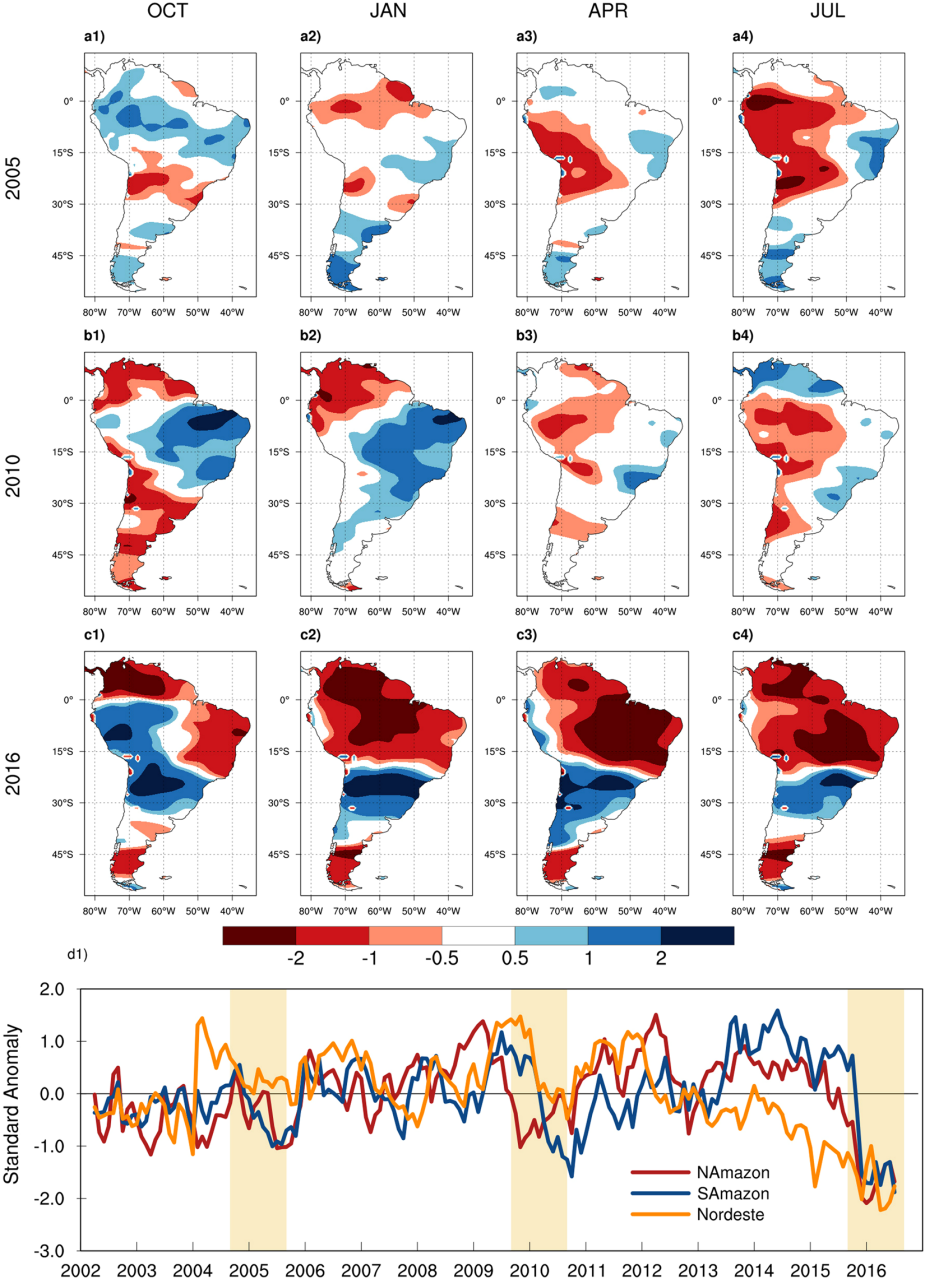
Spatial maps and time series of TWS standardized anomalies calculated using GRACE data from 2002 to 2017. The contour plots present the seasonal anomalies for the three extreme droughts of 2005 (**a1,a2,a3,a4**), 2010 (**b1,b2,b3,b4**), and 2016 (**c1,c2,c3,c4**). The monthly time series (**d1**) were constructed by averaging anomalies over the North Amazon, South Amazon, and Nordeste regions and the highlight strips denote the extreme droughts represented in the spatial maps. Seasonal cycles for each year start from September of the previous year (e.g. SON 2004 for 2005 seasonal cycle). Figures created with: The NCAR Command Language (Version 6.3.0) [Software]. (2016). Boulder, Colorado: UCAR/NCAR/CISL/TDD. http://dx.doi.org/10.5065/D6WD3XH5.

Standardized anomalies of GRACE TWS during the three most recent extreme droughts are presented in Fig. 3 as a metric for the cumulative effects of precipitation anomalies on terrestrial hydrology. The anomalies indicate large decreases of TWS in South Amazon during the dry seasons (MAM and JJA) of 2005 and 2010. For the 2016 drought, the GRACE data indicates strong negative TWS anomalies over the Nordeste region and North Amazon in SON. As the precipitation deficits extend into the dry season, the TWS anomalies become extremely negative over the entire tropical/subtropical South America. The time series of the TWS anomalies averaged over the three regional domains also indicate that the worst TWS deficits over the entire length of GRACE data occurred in 2016. For the Nordeste region in particular, the TWS anomalies have continuously depleted from 2012 to 2017, signifying a long-term extreme drought. Over most of tropical and subtropical South America, the strong TWS deficits persist beyond the end of the 2016 meteorological drought.

**Figure 4:**
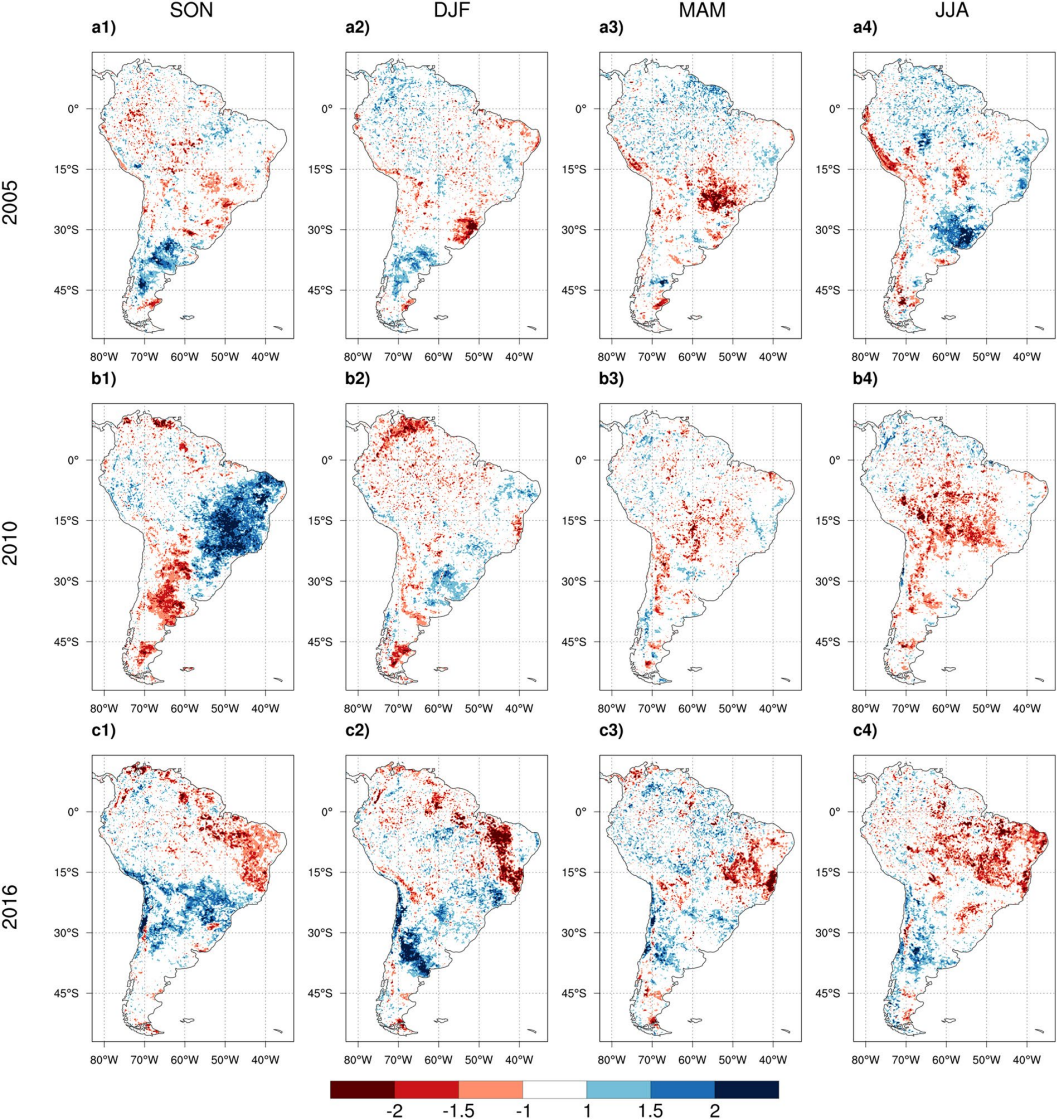
Spatial maps of Normalized Difference Vegetation Index (NDVI) anomalies calculated using NASA MODIS product over 2003–2017. The seasonal anomalies are shown for the three extreme droughts of 2005 (**a1,a2,a3,a4**), 2010 (**b1,b2,b3,b4**), and 2016 (**c1,c2,c3,c4**). Seasonal cycles for each year start from September of the previous year (e.g. SON 2004 for 2005 seasonal cycle). Figures created with: The NCAR Command Language (Version 6.3.0) [Software]. (2016). Boulder, Colorado: UCAR/NCAR/CISL/TDD. http://dx.doi.org/10.5065/D6WD3XH5.

To investigate the drought impact on vegetation, we used NDVI and EVI as proxies for vegetation greenness (Fig. 4 and Figure S5). During the 2015–2016 event, substantial decreases in vegetation greenness were observed during the SON and DJF seasons over northeastern Amazon, in all four seasons over the Nordeste region, and across most of tropical and subtropical South America during JJA (Fig. 4). The extensive areas with large negative NDVI or EVI in 2016 in the Nordeste region and eastern Amazon also distinguish the recent event from the two previous droughts of 2005 and 2010, which were centered over central and southern Amazon. Overall, the dry season (JJA) stands out for its extent of severe decrease of greenness during both the 2010–2011 and 2015–2016 events. Evidently, the three events differ in the extent, severity, and location of the negative NDVI anomalies, with the strongest decrease of vegetation greenness observed during the 2015–2016 drought. It is important to note that extra caution should be taken when using a satellite-based greenness index as a proxy for vegetation response to drought as use of different products has led to contradictory conclusions in the literature^14, 34, 35^. The data used here is extracted from the latest version of the MODIS products which was cloud-filtered and corrected for aerosol/atmosphere corruption effects (see methodology).

For drought prediction, a statistical multivariate linear regression model was developed to estimate precipitation anomalies based on oceanic forcings in both the Pacific and Atlantic basins. Performance of the regression model and its predictions are presented in Figure S6. In general, SST anomalies are better predictors for rainfall anomalies over North Amazon than South Amazon and the Nordeste region. The spatial maps for coefficients of determination (Figure S6) indicate that the regression model can reproduce more than 70% of precipitation variance over the Northern Amazon, the Northern area of the Nordeste region and much of the La Plata basin for all four seasons. Among the five highlighted extreme droughts, the 1983 and 1998 events are included in the calibration period (1982–2002), and the three more recent events are included in the verification period (2003–2017). Compared to the observed rainfall anomalies shown in Fig. 2, the model reproduces the spatial patterns and magnitudes of the SON and DJF precipitation anomalies for 2016 reasonably well over North Amazon, but substantially underestimates them over South Amazon and Nordeste. Rainfall deficits in MAM and JJA of 2016 over Nordeste are underestimated as well. For the 2005 and 2010 events, the model-produced anomalies are comparable with observations, except for a slight underestimation over South Amazon. For the El Niño-induced droughts of 1983, 1998 and 2016, the regression model underestimates the dry season (MAM and JJA) rainfall anomalies over tropical South America to a degree that differs among events (Figure S6).

**Figure 5:**
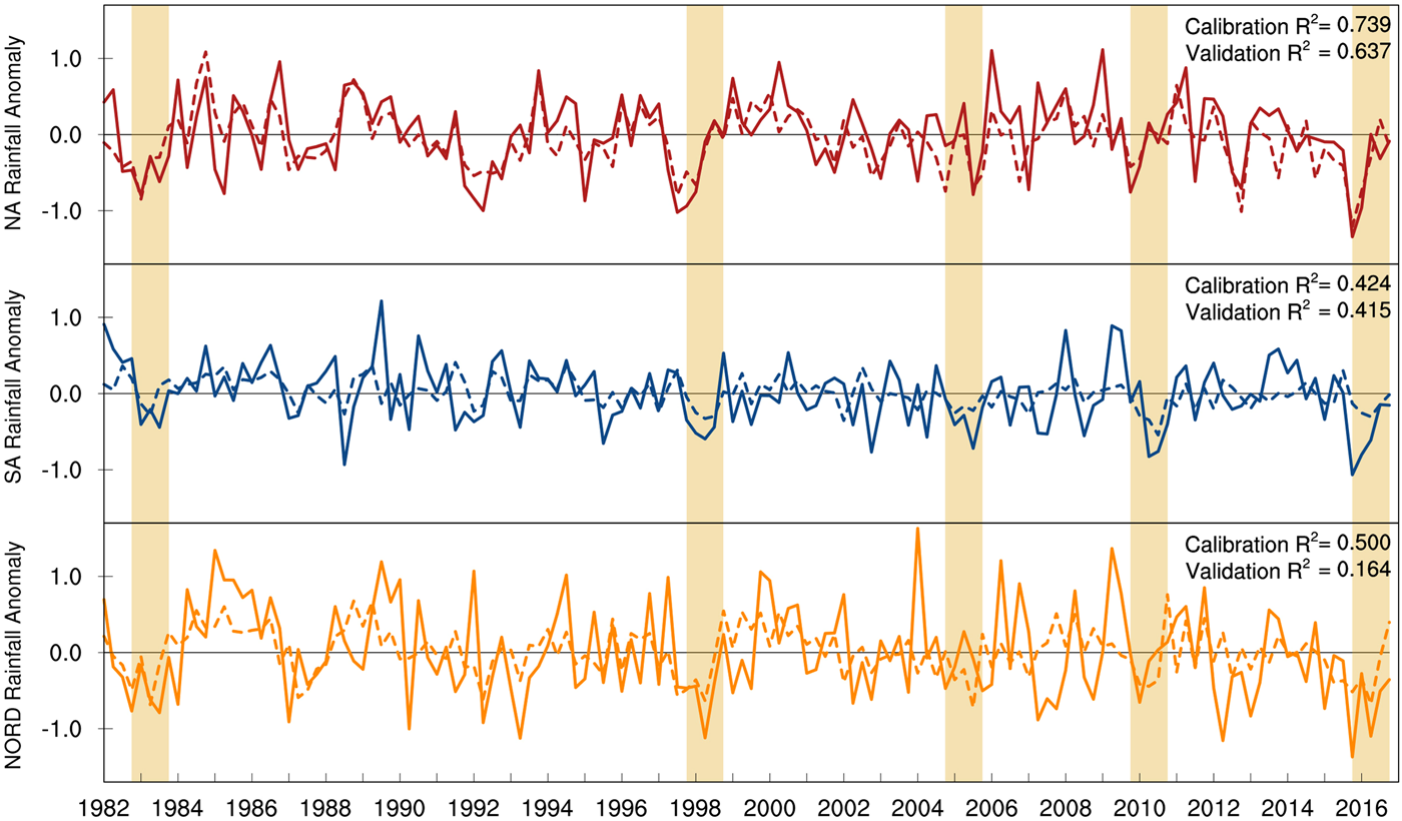
Time series of the predicted (dashed lines) versus observed (solid lines) seasonal precipitation anomalies over North Amazon (top), South Amazon (middle), and Nordeste (bottom). The predicted and observed time series of rainfall deficits are used to calculate coefficient of determination (R^2^) for the calibration (1982–2001) and validation (2002–2017) periods. Compared to South Amazon and Nordeste, the predicted anomalies explain the rainfall variability better over North Amazon. The model prediction for 2016 rainfall deficits closely resemble the observations over North Amazon yet, substantially underestimate the deficits observed over South Amazon and Nordeste. Figures created with: The NCAR Command Language (Version 6.3.0) [Software]. (2016). Boulder, Colorado: UCAR/NCAR/CISL/TDD. http://dx.doi.org/10.5065/D6WD3XH5.

The spatial average of precipitation anomalies predicted by the multivariate linear regression model are compared with observations in Fig. 5. Over North Amazon, the model can explain 74% of the observed variance over the calibration period and 64% over the verification period, and the model rainfall deficits for the five highlighted extreme droughts closely match observations. Over South Amazon, the model accounts for about 42% of the observed variance for both the calibration and verification periods. Precipitation anomalies are slightly underestimated for most of the highlighted extreme events, yet the 2016 drought is substantially underestimated over this region. Over Nordeste, the model explains 50% of the observed variance during the calibration period, but only 16% of the observed variance for the verification period. The regression model performs reasonably well in reproducing the rainfall deficits of 1982, 1998 and 2010 over Nordeste, but substantially underestimates the severity of 2016 (and 2007 and 2012) rainfall deficits.

**Figure 6:**
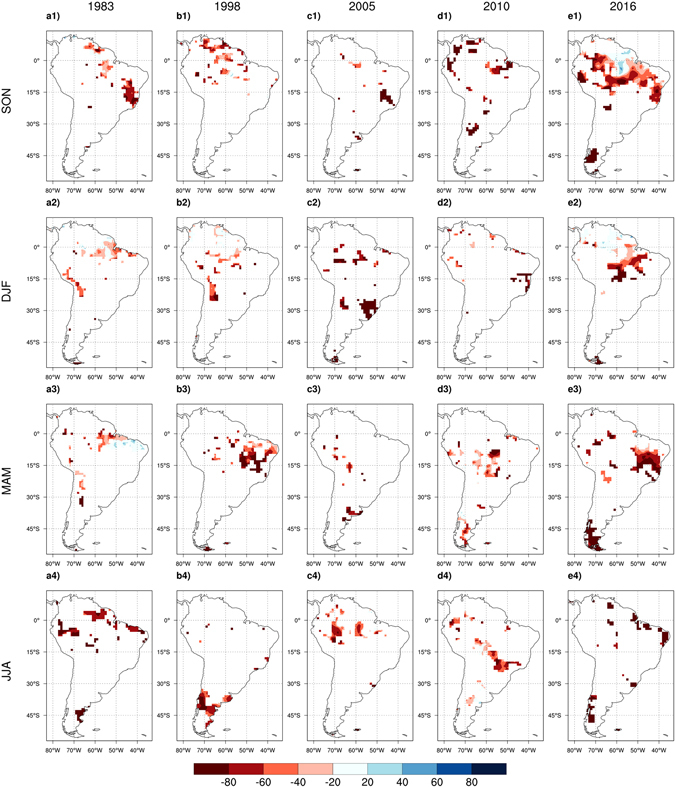
Spatial maps of model relative biases in predicting the magnitude of precipitation anomalies for the five extreme droughts of 1983 (**a1** to **a4**), 1998 (**b1** to **b4**), 2005 (**c1** to **c4**), 2010 (**d1** to **d4**), and 2016 (**e1** to **e4**). The areas where magnitude of the observed negative standardized anomalies of precipitation is lower than 1.0 are masked out in order to only focus on regions that experience intense drought. Here, the least-square fit is implemented over the entire analysis period (1982–2017) to equally account for all five extreme droughts when deriving the regression coefficients of the multivariate linear model. Seasonal cycles for each year start from September of the previous year (e.g. SON 2004 for 2005 seasonal cycle). Figures created with: The NCAR Command Language (Version 6.3.0) [Software]. (2016). Boulder, Colorado: UCAR/NCAR/CISL/TDD. http://dx.doi.org/10.5065/D6WD3XH5.

Figure 6 presents the spatial pattern of model relative biases in predicting the precipitation anomalies of the five highlighted events, focusing on areas of extreme drought (where the observed precipitation is more than one standard deviation below the long-term mean). The model performs remarkably well in predicting the extreme droughts for the first four events suggesting strong predictability of these droughts from the oceanic drivers. The 2016 drought, however, is an exception as the model substantially under-predicts the drought severity over an extensive region of the Amazon and Nordeste. The strong underestimation signifies an important distinction between the underlying drivers of the extreme rainfall deficits of the 2016 event and those of the previous events.

Outlier analysis is implemented over the regression results to measure the significance of the deviation of precipitation anomalies from the long-term SST-rainfall relationship. Here, outliers represent anomalous precipitation in any specific season that are highly unlikely to be predicted by the empirical model. Figure S7 presents the spatial maps of the outlier residuals for the five extreme droughts of interest where the residuals pass the 10% significance level (one-sided T distribution with df = 29). Compared to the 2016 drought, the areal extent of the outlier residuals is relatively small for the 1983, 1998, 2005, and 2010 events. For the 2016 event, the extent of the areas with extremely large residuals increases substantially. The RMSEs calculated for the (negative) standardized residuals presented in each panel signify that the magnitudes of the outlier residuals are largest for the 2016 event in comparison with the other four extreme events.

## Conclusions and Discussion

The SPI index and precipitation standardized anomalies suggest that the severity and extensiveness of the 2016 meteorological drought in the Amazonian and Nordeste regions is the greatest in our study period. The 2016 event surpasses the severity of the 2005 and 2010 droughts, both considered 100-year events. The most recent drought was driven primarily by anomalously warm tropical Pacific (El Niño) SSTs whereas the previous droughts of 2005 and 2010 were mainly caused by anomalously warm tropical Atlantic SSTs. The SPIs calculated for both short term and long term timescales over the entire GPCC data length (1901–2017) confirm that the severity of the 2016 drought is unprecedented. Comparing the rainfall anomalies of GPCC with TRMM (Figure S8) also indicates that the results are not sensitive to the precipitation data used in our analysis. Note that results of the extended precipitation analysis should be treated with caution due to potentially lower data quality in the early twentieth century.

Among the contributing sources, variability of the tropical oceans is the main driver of inter-annual rainfall variability over the region. For the past decade, the historical SST-rainfall relationship, without including other factors, was able to account for 65% of the rainfall variance over North Amazon and 40% over South Amazon, but only 15% over the Nordeste region. For the 2016 drought, the Nordeste region and Southeastern Amazon are identified as outlier areas where observed rainfall anomalies are highly unlikely to be explained by the oceanic indices considered in the model. These results signify the distinctive nature of the 2016 event as compared to the previous extreme droughts emphasizing an imperative need to study the underlying mechanisms of the most recent event.

Although oceanic variability is still the main driver of droughts in the region, contribution of other drivers has yet to be investigated. Disturbances and other processes over land, including deforestation, surface warming, and CO2 fertilization, are shown to influence the regional hydroclimate regime of tropical South America. Significant warming and drying trends have been observed over the region during the past decade^36–39^. Greenhouse gas warming tends to intensify droughts over the sub-tropics in response to the anticipated poleward expansion of the Hadley Cell in a warmer climate^40^. Surface warming can also exacerbate the hydrological and agricultural consequences of precipitation deficits by accelerating evapotranspiration and depleting soil moisture. For the 2016 drought, a recent study suggested that regional surface warming might have intensified the drought severity^17^. Numerous studies have suggested that anthropogenic land use changes such as forest loss can cause warming and precipitation deficits in the Amazon^30, 31, 41–43^. A newly published study indicated that deforestation in Amazonia has advanced enough to cause a shift from a thermally to dynamically driven hydroclimatic regime, reducing precipitation over the deforested areas^44^. On the role of vegetation dynamics in shaping precipitation variability in the Amazon region, Wang *et al*.^45^ suggested that “many areas of the Amazon will be prone to recurrent droughts during the several years following the 2010 drought” due to the slow recovery of the drought-stressed vegetation. Indeed, multiple droughts have been reported since then^17, 46^, including a severe drought in 2012–2013^10^ (also see Fig. 2-e1) though it did not rival the 2016 event in severity. Mounting evidence from the literature, along with the rarity of the 2016 drought in long term observations and unpredictability of its intensity from the SST-rainfall relationship, emphasize the importance of investigating the contribution of anthropogenic and terrestrial processes in amplifying droughts in this region. Such investigations could be facilitated by conducting thorough event-based detection and attribution analysis using large ensembles of climate simulations^47^.

## Methods

### Data

We used two different precipitation datasets in order to ensure that our conclusions were not sensitive to uncertainties in observations: Gauge-based monthly precipitation data from Global Precipitation Climatology Center (GPCC) version 7 (1° × 1°), and satellite-based monthly precipitation estimates from the Tropical Rainfall Measuring Mission (TRMM) 3B43 product (0.25° × 0.25°). TRMM data span from 1998 to present and GPCC data are available from 1901 to present. We also used monthly mean SSTs from the NOAA Optimum Interpolation Sea Surface Temperature (OISST)^48^ data set version II (1° × 1°) which spans October 1981 to the present. To be consistent with SST data coverage, we used GPCC rainfall data from 1982 to 2017. Comparison of the two precipitation data sets was done for their overlapping period 1998–2016 (see Figure S8). To derive the monthly standardized anomalies for both rainfall and SST, we first subtracted the climatological seasonal cycle from monthly data and then normalized the anomalies by the standard deviation of each month.

Monthly anomalies of TWS were derived from Gravity Recovery and Climate Experiment (GRACE) data^27^. The GRCTellus land grid data represent deviation of the surface mass in each month from the average mass of the baseline period (Jan 2004 to Dec 2009). The grid data set used here has a 1° × 1° spatial resolution and is publicly available at NASA JPL website (https://grace.jpl.nasa.gov/data/get-data/monthly-mass-grids-land). To remove the attenuation of the surface mass variations at small spatial scales, the land grid scaling has been applied to the raw GRACE data fields as suggested by ref. 28. The scaled data, in units of centimeters of equivalent water thickness, were then used to construct the standardized anomalies (dimensionless) over the period of 2002 to 2016.

NDVI and EVI were derived from the MOD13C2 product of the Moderate Resolution Imaging Spectroradiometer (MODIS) sensors aboard Terra and Aqua satellites^49^. The MOD13C2 is cloud-free monthly data on a 0.05 degree (5600 meters) geographic Climate Modeling Grid (CMG) which is constructed from the gridded 16-day 1-kilometer MOD13A2 data^49^. The data is available from July 2002 to near real-time present day and can be downloaded at the NASA-USGS Land Process Distributed Active Archive Center (LP DAAC) website (https://lpdaac.usgs.gov/dataset_discovery/modis/modis_products_table/mod13c2). The standardized anomalies are constructed by subtracting the monthly climatology (calculated over 2003–2017) from the monthly data and dividing the anomalies by the standard deviation for each month. The vegetation indices use the wavelength and intensity of the reflected light within the visible and near-infrared wavelengths to measure density of green leaf vegetation. Both NDVI and EVI values span the −1 to +1 range where very low values (<0.1) represent barren land with no green leaf and +1 indicates the highest possible density of green leaves.

### SPI

To measure the severity of meteorological droughts, we used the Standardized Precipitation Index (SPI)^50^ with an averaging period of three, six, twelve, and twenty-four months. Historical time series of monthly precipitation for each grid cell were used to extract the shape (α) and scale (β) factors of a (two-parameter) Gamma distribution function using maximum likelihood estimate. The calculated SPI values were then normally distributed and any specific SPI value represents the departure of the corresponding precipitation from the long-term mean normalized by its standard deviation. To construct the seasonal SPI maps, we averaged the monthly SPI values of corresponding seasons. Two data periods were used in our SPI analysis. The 1982–2017 period was used as the base period of SPI (and regression analysis) which was selected based on availability of the high quality observed data for SST and rainfall. The 1901–2017 time period was also used in SPI analysis to investigate sensitivity of the 1982–2017 SPIs to the data length.

### Empirical prediction model

An empirical prediction model was developed to predict the rainfall anomalies from the SST anomalies of the tropical Pacific, North Atlantic, and South Atlantic. The empirical models using long-term rainfall and SST observations were shown to successfully explain most of Amazon and Nordeste precipitation variability^8, 19, 21, 29^, enabling skillful prediction of rainfall deficits and wildfire risk^8, 12, 19^.

The model is a linear regression model between time series of precipitation standardized anomalies at grid cell (i, j), P(i, j, t), with those of area-averaged tropical SST anomalies at Pacific (Nino3.4), North Atlantic (NAT) and South Atlantic (SAT) regions (see Figure S1 for denotation of the Oceanic basins over map):

$${\boldsymbol{P}}({\boldsymbol{i}},{\boldsymbol{j}},{\boldsymbol{t}}){\boldsymbol{=}}{{\boldsymbol{\beta }}}_{0}({\boldsymbol{i}},{\boldsymbol{j}}){\boldsymbol{+}}{{\boldsymbol{\beta }}}_{1}({\boldsymbol{i}},{\boldsymbol{j}}){\boldsymbol{\times }}{\boldsymbol{Nino}}{\bf{3.4}}({\boldsymbol{t}}){\boldsymbol{+}}{{\boldsymbol{\beta }}}_{2}({\boldsymbol{i}},{\boldsymbol{j}}){\boldsymbol{\times }}{\boldsymbol{NAT}}({\boldsymbol{t}}){\boldsymbol{+}}{{\boldsymbol{\beta }}}_{3}({\boldsymbol{i}},{\boldsymbol{j}}){\boldsymbol{\times }}{\boldsymbol{SAT}}({\boldsymbol{t}})\,$$

where, ***β***_**1**_, ***β***_**2**_, ***β***_**3**_ are spatially varying coefficients determining sensitivity of precipitation to the individual oceanic drivers for each grid cell and ***β***_**0**_ is the regression constant. All the regression coefficients were calculated using least square fitting of GPCC seasonal precipitation time series from 1982 to 2002 with seasonal time series of SST indices for the same period.

### Outlier Analysis

Residuals in least square linear models are used to investigate the appropriateness of regression models and to detect the extreme observations or outliers in the space of predictand (y or dependent variable)^51, 52^. Here we used the externally studentized residual approach for outlier analysis. The regular residuals in a regression model, ${e}_{i}=\,{y}_{i}-\,\widehat{{y}_{i}}$ have different variances that vary based on the predictor(s). The variance of the i_th_ residual is calculated using the following equation^52^:

1 $${\sigma }_{{e}_{i}}^{2}={\sigma }^{2}(1-{h}_{i})$$

where *σ* ^2^ is variance of the raw residuals, ${\sigma }_{{e}_{i}}^{2}$ is the variance of the i_th_ residual, and h_i_ is the leverage and is the i_th_ diagonal element of the hat matrix (H). The studentized residuals are derived by normalizing the raw residual by their estimated standard deviation:

2 $${r}_{i}=\frac{{e}_{i}}{{\sigma }_{{e}_{i}}}=\frac{{e}_{i}}{\sigma \sqrt{1-{h}_{i}}}$$

The studentized residuals are called externally studentized if *σ* in eq. 2 is replaced with *σ*_−*i*_, which is the standard deviation of the residuals for a regression fitted over all the data excluding the i_th_ point.

The studentized residuals then can be used to calculate the t statistics from the following equation to test the outliers for significance^53^:

3 $${t}_{i}={r}_{i}\sqrt{\frac{n-p-1}{n-p-{r}_{i}^{2}}}$$

Here p is the number of regression parameters (including the regression constant), n is the sample size, and *t*_*i*_ is t statistic of the i_th_ residual for the T distribution with n-p-1 degree of freedom. The externally studentized residuals allow us to conduct “case analysis” by isolating each individual data point and measuring the likelihood of that point being erroneous to the linear model that is fitted over the rest of the points in the data set.

## Electronic supplementary material

Supplementary material
