# Supplementary material for: Unprecedented drought over tropical South America in 2016: significantly under-predicted by tropical SST
Source: Sci Rep. 2017 Jul 19;7:5811. doi: 10.1038/s41598-017-05373-2 (PMC5517600; doi:10.1038/s41598-017-05373-2)
Supplement: Supplementary file 1 — Supplementary material [file 41598_2017_5373_MOESM1_ESM.doc]

# Unprecedented drought over tropical South America in 2016: significantly under-predicted by tropical SST

Amir Erfanian^1^, Guiling Wang^1*^, Lori Fomenko^1^

# Supplementary material

^1^Department of Civil and Environmental Engineering and Center for Environmental Sciences and Engineering, University of Connecticut, Storrs, Connecticut, USA

* Correspondence:

Dr. Guiling Wang

Department of Civil and Environmental Engineering

University of Connecticut

Storrs, CT, 06268-3037

Email: [guiling.wang@uconn.edu](mailto:guiling.wang@uconn.edu)

Phone: 860 486 5648

Figure S1- Areal representation of the region defined over South America as well as the oceanic regions used in calculating Nino3.4 [1], Tropical North Atlantic (TNA) and Tropical South Atlantic (TSA) indices. North Amazon spans from 75W to 50W and 5S to 7N [2], South Amazon spans from 75W to 50W and 5S to 17S [2], and Nordeste spans from 50W to 34W and 17S to 0°. Nino3.4 is defined between 150W to 90W and 5S to 5N, TNA spans from 57.5W to 15W and 5.5N to 23.5N, TSA spans from 30W to 10E and 20S to 0° [3]. Figures created with: The NCAR Command Language (Version 6.3.0) [Software]. (2016). Boulder, Colorado: UCAR/NCAR/CISL/TDD. http://dx.doi.org/10.5065/D6WD3XH5.


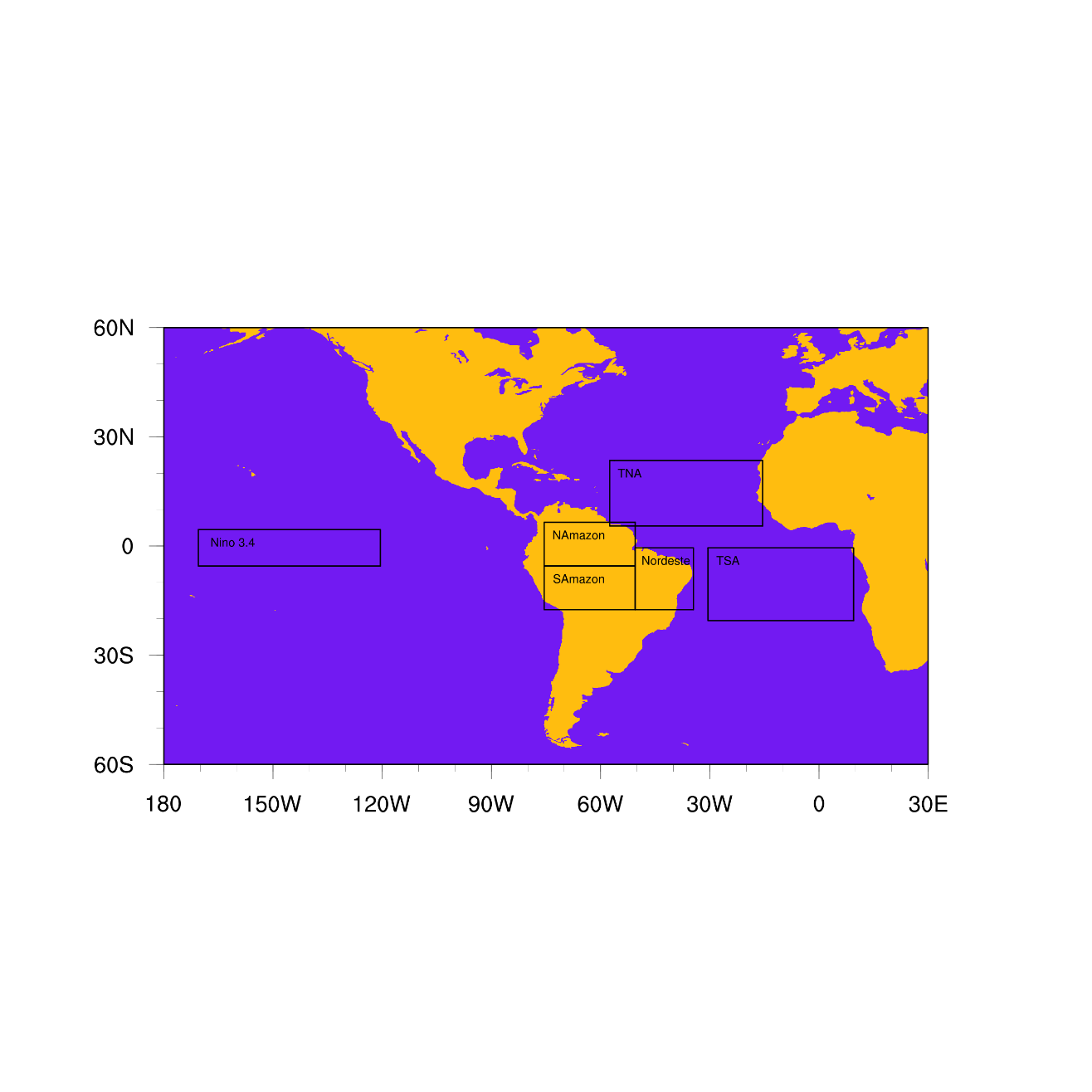


Figure S2 – Same as Figure 1 but the SPIs are calculated using the GPCC precipitation data from 1901 to 2017. Figures created with: The NCAR Command Language (Version 6.3.0) [Software]. (2016). Boulder, Colorado: UCAR/NCAR/CISL/TDD. http://dx.doi.org/10.5065/D6WD3XH5.


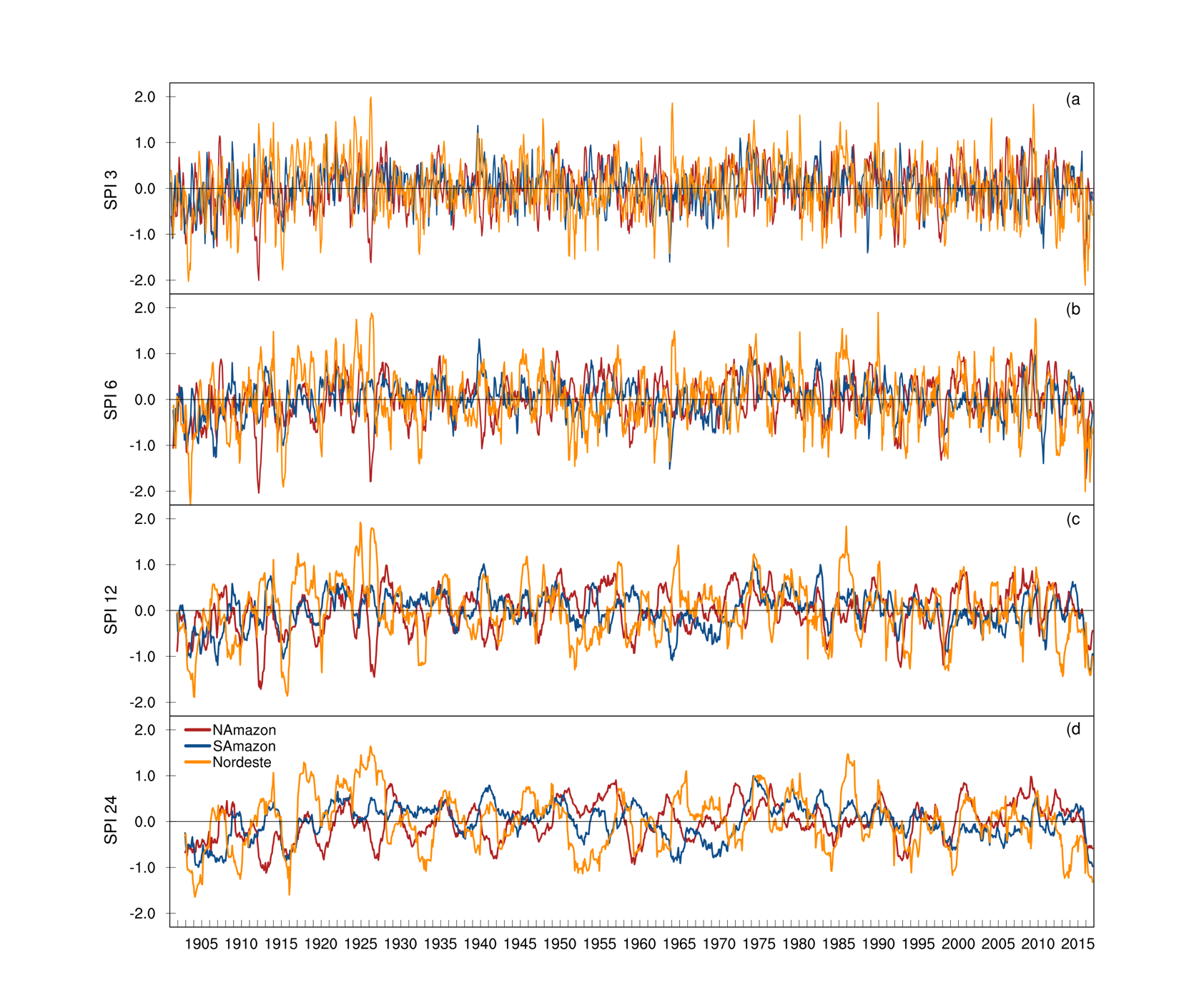


Figure S3- Same as Figure 2 but for 3-month SPI. Figures created with: The NCAR Command Language (Version 6.3.0) [Software]. (2016). Boulder, Colorado: UCAR/NCAR/CISL/TDD. http://dx.doi.org/10.5065/D6WD3XH5.


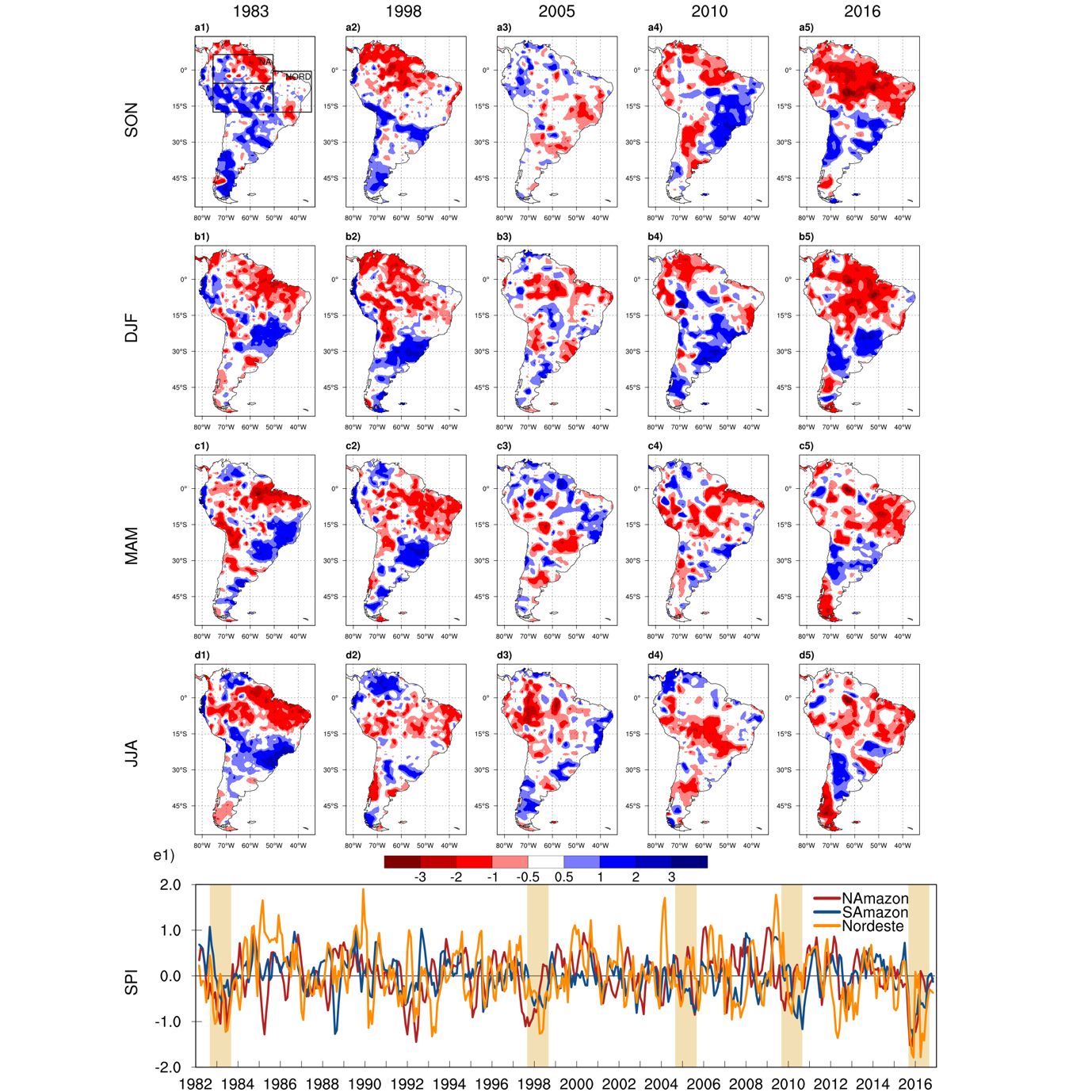


Figure S4- SST standardized anomalies calculated using OISST monthly mean data from 1982 to 2017. Seasonal anomalies for each year start from September of the previous year (e.g. SON 2004 for the 2005 cycle). The time series of monthly anomalies (e) are constructed for the three oceanic basins (see Figure S1 for detailed specification of the oceanic indices) by doing the spatial averaging first, and calculating standardized anomalies next. Spatial maps of seasonal SST anomalies for 2005 (a1,b1,c1,d1), 2010 (a2,b2,c2,d2), and 2016 (a3,b3,c3,d3) indicate different patterns of tropical SST anomalies for the three extreme droughts. The distinct patterns of Tropical SST anomalies result in different seasonality for the three extreme droughts. Figures created with: The NCAR Command Language (Version 6.3.0) [Software]. (2016). Boulder, Colorado: UCAR/NCAR/CISL/TDD. http://dx.doi.org/10.5065/D6WD3XH5.
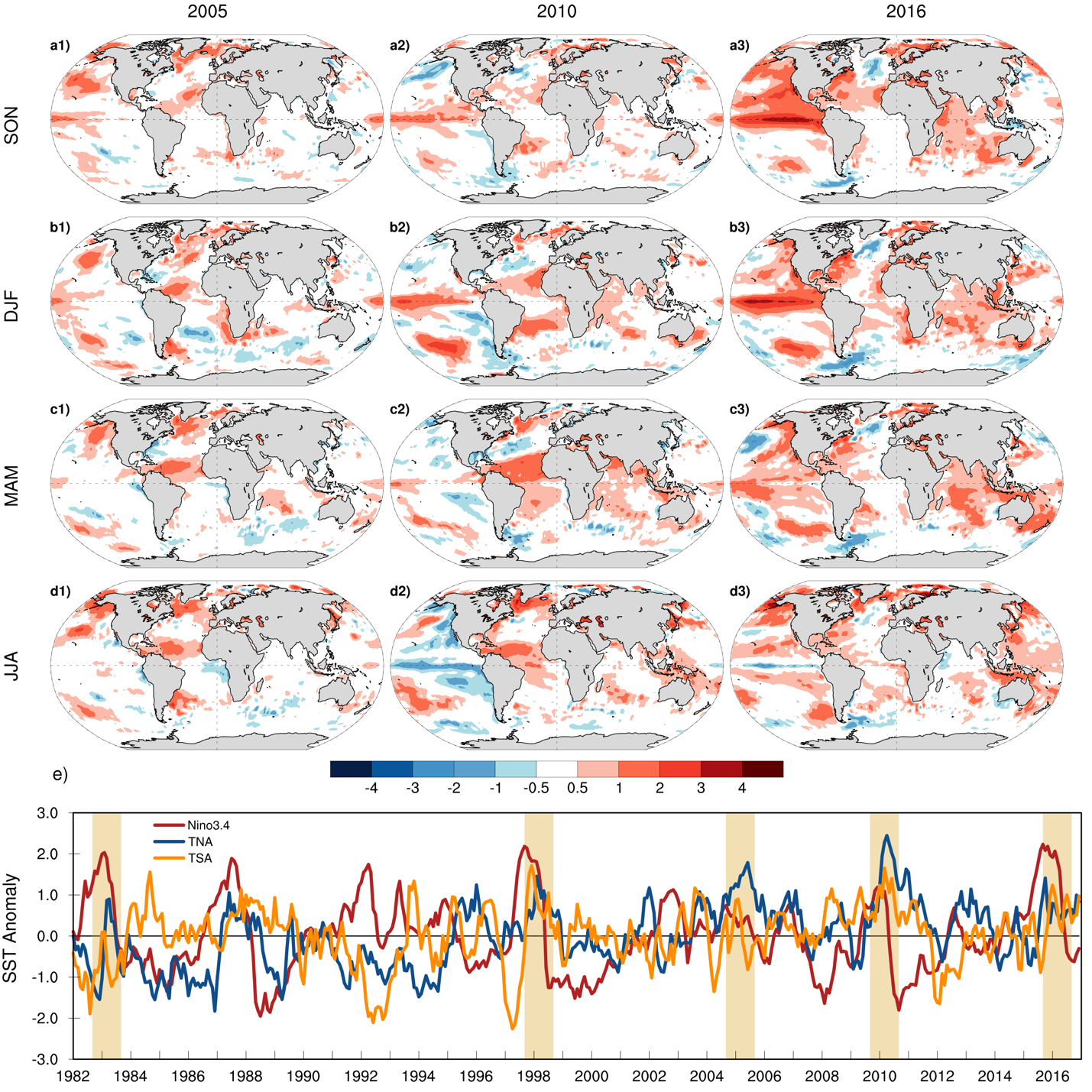


Figure S5- Same as Figure 4 but for the Enhanced Vegetation Index (EVI). Figures created with: The NCAR Command Language (Version 6.3.0) [Software]. (2016). Boulder, Colorado: UCAR/NCAR/CISL/TDD. http://dx.doi.org/10.5065/D6WD3XH5.


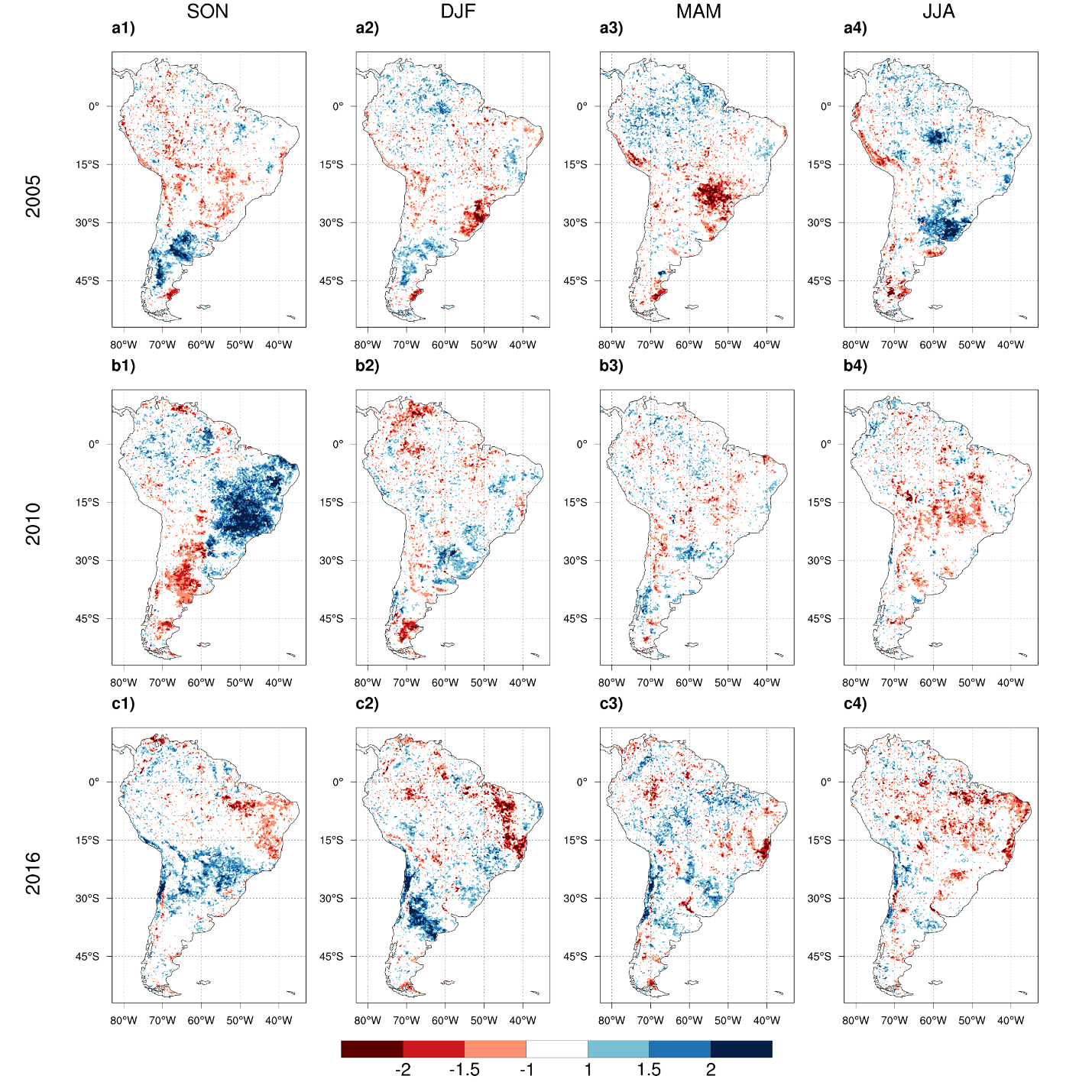


Figure S6 - Empirical prediction of precipitation deficits over South America. The spatial maps present the rainfall anomalies predicted from the regression model during 1983 (a1 to a4), 1998 (b1 to b4), 2005 (c1 to c4), 2010 (d1 to d4), and 2016 (e1 to e4). The regression coefficients are calculated for each grid cell at a seasonal time scale where seasonal anomalies of each year start from September of the previous year (e.g. SON 2004 for 2005 seasonal cycle). The predicted and observed time series of rainfall deficits during the calibration period (1982-2001) are used to calculate coefficient of determination (R^2^) for each season at each grid cell (f1 to f4). Figures created with: The NCAR Command Language (Version 6.3.0) [Software]. (2016). Boulder, Colorado: UCAR/NCAR/CISL/TDD. http://dx.doi.org/10.5065/D6WD3XH5.


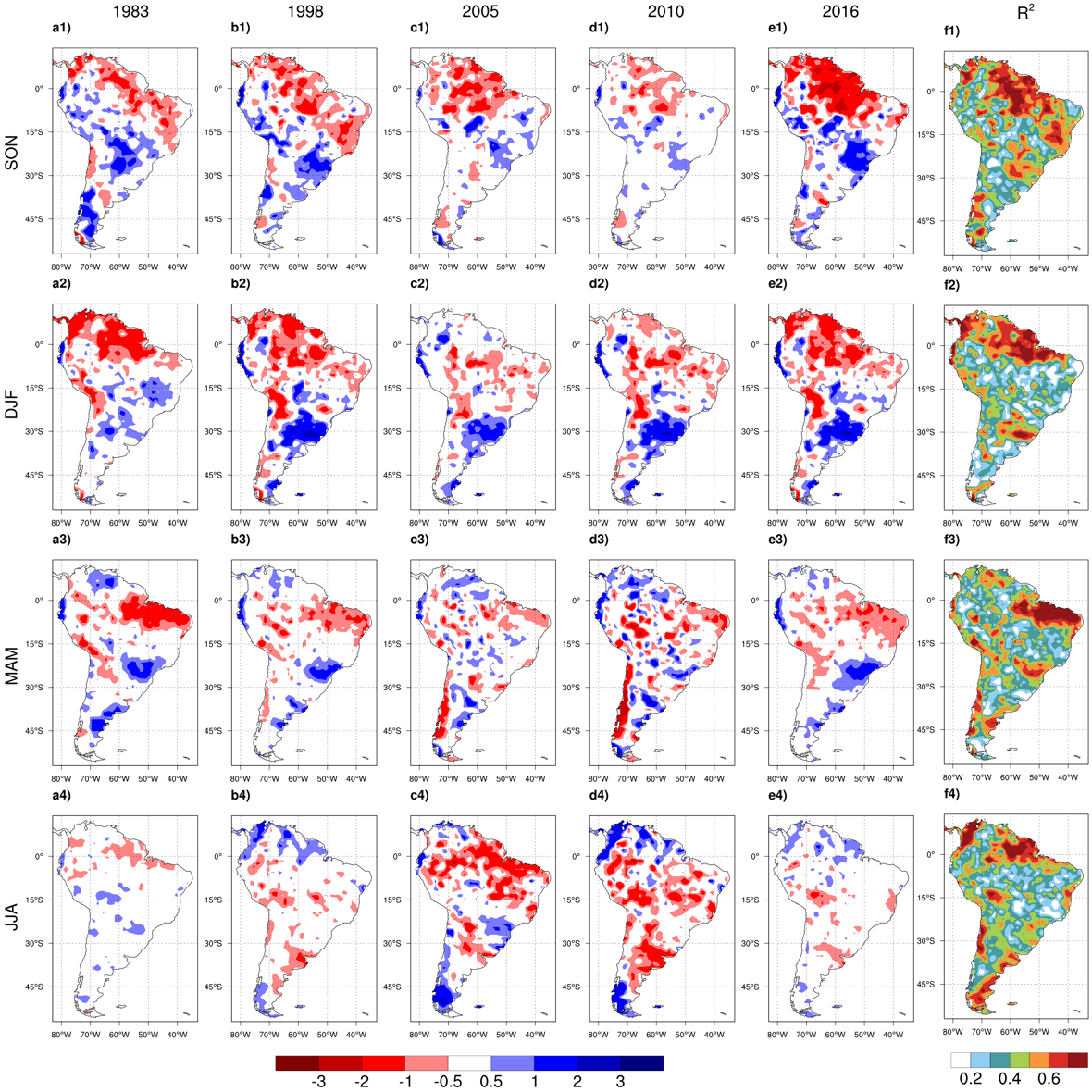


Figure S7 – Analysis of the residuals (observation – prediction). The contour plots present the externally studentized residuals for the five extreme droughts of 1983 (a1,a2,a3,a4), 1998 (b1,b2,b3,b4), 2005 (c1,c2,c3,c4), 2010 (d1,d2,d3,d4), and 2016 (e1,e2,e3,e4). The color bar is scaled to represent the t values corresponding to 95% (±1.7), 90% (±1.3), and 85% (±1.0) one-sided probability levels from the T distribution with 29 degree of freedom. The seasonal cycles in each year start from September of the previous calendar year (e.g. SON 2004 for 2005 seasonal cycle). The RMSE values in each panel are calculated only using the negative residuals. Figures created with: The NCAR Command Language (Version 6.3.0) [Software]. (2016). Boulder, Colorado: UCAR/NCAR/CISL/TDD. http://dx.doi.org/10.5065/D6WD3XH5.


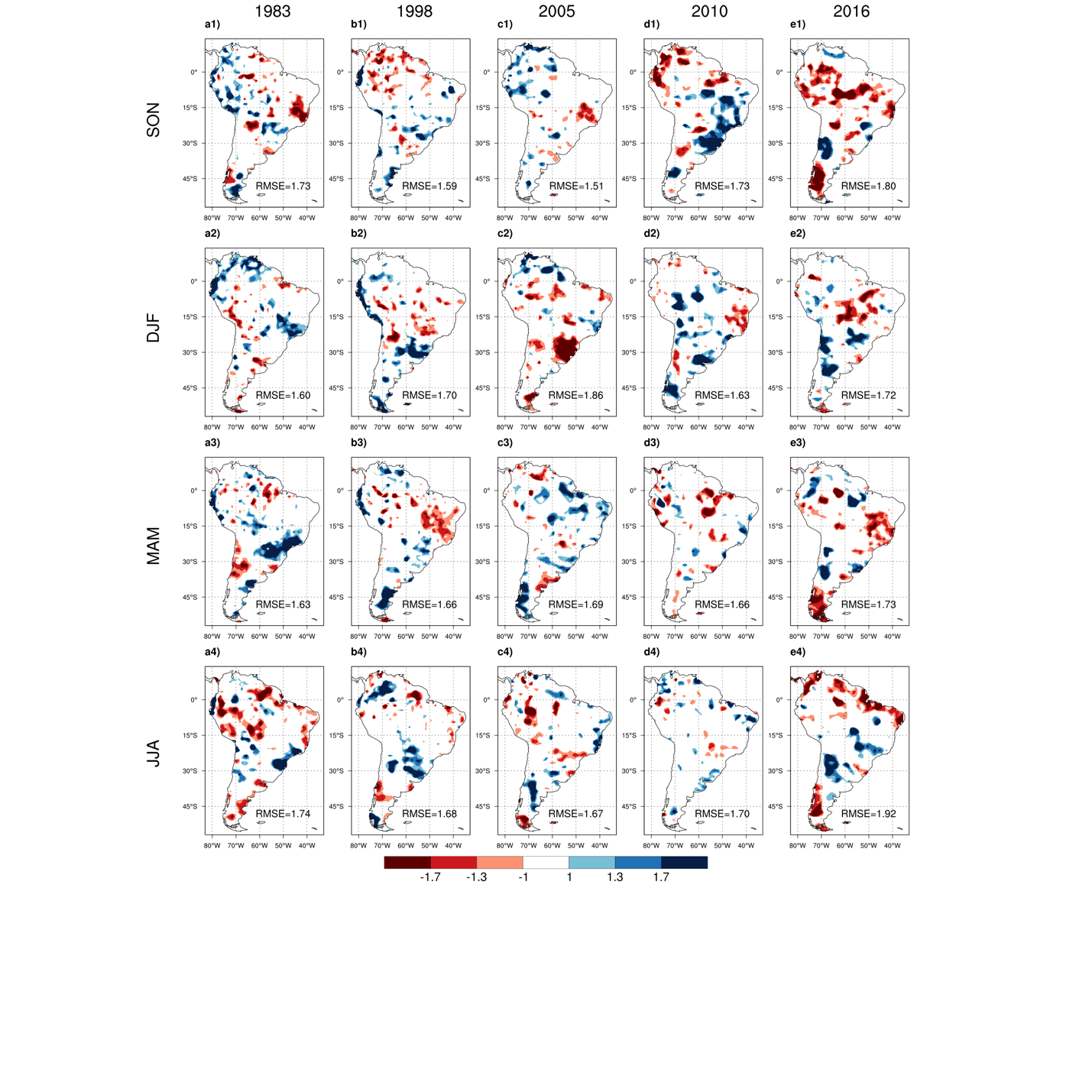


Figure S8- Standardized precipitation anomalies (dimensionless) calculated for GPCC and TRMM(B43B) products over 1998 to 2017. The comparison between the spatial maps of 2016 seasonal anomalies from GPCC (1^st^ row) with those of TRMM (2^nd^ row) indicates that our conclusions about the severity and spatial patterns of the 2016 droughts are independent from the precipitation product. Comparison between the time series of TRMM rainfall (blue lines) and those of GPCC data (red line) over North Amazon (c1), South Amazon (c2), and Nordeste (c3) indicates slightly higher precipitation in DJF (b) and MAM (c) over Western and Northwestern Amazonia as well as eastern Nordeste. As a result, monthly times series of TRMM rainfall anomalies over the corresponding months indicate even more extreme deficits for all the three areas. Figures created with: The NCAR Command Language (Version 6.3.0) [Software]. (2016). Boulder, Colorado: UCAR/NCAR/CISL/TDD. http://dx.doi.org/10.5065/D6WD3XH5.


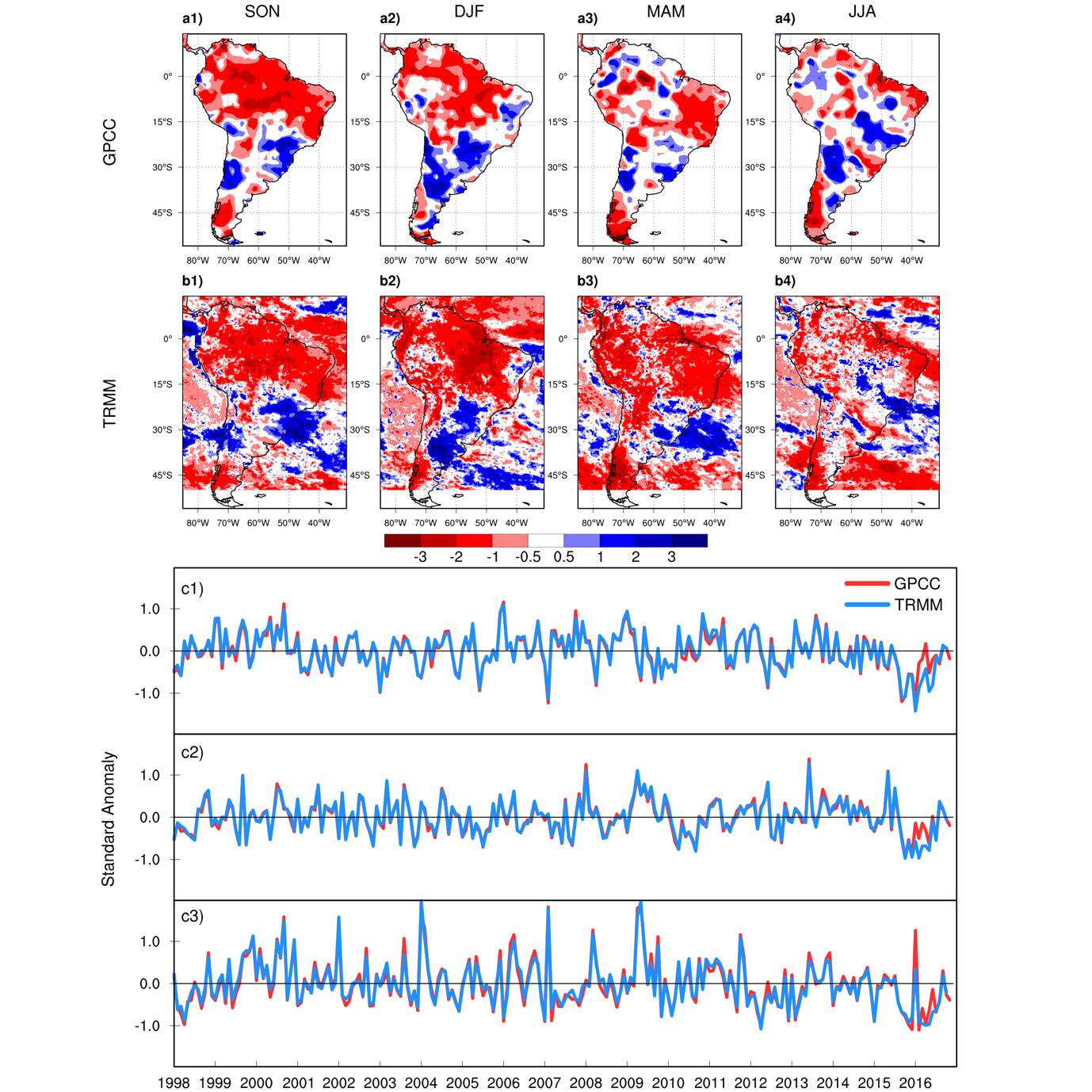


# Reference

1. Trenberth, K. & Stepaniak, D. Indices of El Niño evolution. *Journal of Climate.* **14**, 8, 1697-7101 (2001).

2. Marengo, J., Tomasella, J., Alves, L., Soares, W. & Rodriguez, D. The drought of 2010 in the context of historical droughts in the Amazon region. *Geophysical Research Letters.* **38**, 12, 1-5 (2011).

3. Enfield, D., Mestas‐Nuñez, A., Mayer, D. & Cid‐Serrano, L. How ubiquitous is the dipole relationship in tropical Atlantic sea surface temperatures? *Journal of Geophysical Research: Oceans.* **104**, C4, 7841-7848 (1999).
